# Does genetic diversity protect host populations from parasites? A meta‐analysis across natural and agricultural systems

**DOI:** 10.1002/evl3.206

**Published:** 2020-11-14

**Authors:** Amanda Kyle Gibson, Anna E. Nguyen

**Affiliations:** ^1^ Department of Biology University of Virginia Charlottesville Virginia 22904

**Keywords:** Crop disease, genetic diversity, host heterogeneity, host mixtures, host‐parasite interactions, infectious disease, meta‐analysis, monoculture effect

## Abstract

If parasites transmit more readily between closely related hosts, then parasite burdens should decrease with increased genetic diversity of host populations. This important hypothesis is often accepted at face value—notorious epidemics of crop monocultures testify to the vulnerability of host populations that have been purged of diversity. Yet the relationship between genetic diversity and parasitism likely varies across contexts, differing between crop and noncrop hosts and between experimental and natural host populations. Here, we used a meta‐analytic approach to ask if host diversity confers protection against parasites over the range of contexts in which it has been tested. We synthesized the results of 102 studies, comprising 2004 effect sizes representing a diversity of approaches and host‐parasite systems. Our results validate a protective effect of genetic diversity, while revealing significant variation in its strength across biological and empirical contexts. In experimental host populations, genetic diversity reduces parasitism by ∼20% for noncrop hosts and by ∼50% for crop hosts. In contrast, observational studies of natural host populations show no consistent relationship between genetic diversity and parasitism, with both strong negative and positive correlations reported. This result supports the idea that, if parasites preferentially attack close relatives, the correlation of genetic diversity with parasitism could be positive or negative depending upon the potential for host populations to evolve in response to parasite selection. Taken together, these results reinforce genetic diversity as a priority for both conservation and agriculture and emphasize the challenges inherent to drawing comparisons between controlled experimental populations and dynamic natural populations.

**Impact Summary**

We conducted a large meta‐analysis to test the long‐standing hypothesis that genetic diversity limits parasitism in host populations. We find support for this hypothesis as well as clear evidence that the protective effect of genetic diversity varies across contexts. In experimental host populations, we find that genetic diversity leads to reductions in parasitism that are moderate for noncrop hosts and very strong for crop hosts. These results support a key assumption of major evolutionary hypotheses that argue for parasites as the selective force maintaining sexual reproduction and multiple mating. They also emphasize genetic diversity as a sustainable solution for suppressing disease in crop fields. In contrast, our study did not find a consistent negative correlation between genetic diversity and parasitism across observational studies of natural host populations. This analysis did, however, suggest a negative relationship between genetic diversity and parasitism in the subset of observational studies that surveyed populations of threatened host species. As a whole, our study underscores the potential for genetic diversity to strongly limit parasitism while highlighting biological and empirical factors that may influence where and when we see a relationship between genetic diversity and parasitism. These findings further emphasize that conserving genetic diversity is critical for defending crops and threatened populations against outbreaks.

Why do some host populations suffer from large parasite burdens, while others do not? This is a central question in the study of host‐parasite interactions, and the answer determines best practices for food security and conservation. A prominent hypothesis argues that genetically diverse host populations experience less parasitism than genetically depauperate ones (Haldane 1949; Jensen 1952). This idea assumes parasites have some genetic specificity for infection. Parasites would then transmit more successfully among genetically similar hosts (King and Lively 2012). This hypothesis earned the name the “monoculture effect” based upon devastating epidemics of crop monocultures (Tozzetti and Goidànich 1952; Borlaug 1958; Elton 1958; Leonard 1969; Adams et al. 1971). Although the protective effect of genetic diversity has taken on the status of “conventional wisdom” (King and Lively 2012), its significance appears to vary (Huang et al. 2012; Ekroth et al. 2019). Here, we challenged the generality of the monoculture effect by testing for variation in the effect of genetic diversity on parasitism across the range of contexts in which it has been examined.

Most tests of the monoculture effect compare parasitism in crop mixtures and monocultures (reviewed in Burdon and Jarosz 1990; Smithson and Lenne 1996; Garrett and Mundt 1999; Mundt 2002; Grettenberger and Tooker 2015). Notably, mixing rice cultivars eliminated the need for fungicides by halving damage by rice blast and doubling the yield of a desirable cultivar (Zhu et al. 2000). In a meta‐analysis of 11 studies on stem rust of wheat, the effect of genetic diversity varied, with mixtures showing both more and less parasitism than monocultures. Nonetheless, mixing wheat cultivars reduced stem rust by a mean of 28% (Huang et al. 2012). Across a broader range of crops, cultivar mixtures produce ∼2‐4% greater yields than monocultures on average (Kiær et al. 2009; Borg et al. 2018; Reiss and Drinkwater 2018). This yield advantage increases when parasites are reported as prevalent, suggesting a reduction in parasitism in mixtures as a causal mechanism. Yet, in spite of the vast and varied literature on this topic, there has not been a general test of the hypothesized role for genetic diversity in reducing parasitism across crop systems.

In noncrop systems, the monoculture effect gained traction as a consideration in conservation (O'Brien and Evermann 1988) and as a hypothesis for the maintenance of polymorphism (Haldane 1949), sex (i.e., the Red Queen hypothesis—Jaenike 1978; Hamilton 1980), and polyandry (Sherman et al. 1998). As in crop studies, some researchers tested if parasitism declined when genetic diversity was increased in experimental host populations (e.g., Alexander 1991; Schmid 1994). Other researchers took the question into natural populations, using molecular markers to test for a negative correlation between parasitism and genetic diversity (e.g., Meagher 1999; Trouvé et al. 2003). A meta‐analysis of 23 noncrop studies showed a moderate reduction in parasitism in high‐diversity populations relative to paired low‐diversity populations (Ekroth et al. 2019), although this effect varied with study setting and parasite type. These recent findings call for a comprehensive analysis of experimental and natural populations of noncrop hosts to identify the settings in which genetic diversity limits parasitism and those where it may not.

We built on these prior syntheses by quantifying the monoculture effect across diverse contexts and testing two key hypotheses that predict variation in the relationship between genetic diversity and parasitism. First, we tested the hypothesis that genetic diversity reduces parasitism more strongly for crop hosts than for noncrop hosts. Crops have limited genetic variation, and breeding practices link parasite resistance to single major‐effect loci (Kennedy and Barbour 1992; Hammond‐Kosack and Jones 1997). Moreover, cultivars are extensively phenotyped, so resistance phenotypes can inform assembly of genetically diverse mixtures. Thus, we expect the benefit of increased genetic diversity to be amplified for crop hosts. The early prominence of the monoculture effect in crops inspired evolutionary biologists to apply similar ideas to noncrop systems (King and Lively 2012), and parallels are frequently drawn between the two realms (e.g., Haldane 1949; Elton 1958; Duxbury et al. 2019). In testing this first hypothesis, we ask to what extent these realms resemble one another—does genetic diversity have a comparable effect on parasitism in wild, noncrop systems?

Second, we tested the hypothesis that surveys of natural host populations detect a weaker mean relationship between genetic diversity and parasitism than tests in experimental host populations. Meagher (1999) put forth this idea in his survey of nematode parasitism of deer mice, among the earliest tests of the correlation between genetic diversity and parasitism in natural populations. He argued that, if genetic diversity limits parasitism, both negative *and* positive correlations can arise between diversity and parasitism in wild populations. Negative correlations would arise when parasites “track levels of [host] diversity that are determined by other factors.” Positive correlations would arise when host populations evolve in response to parasite selection—by attacking relatives within common host lineages (Chaboudez and Burdon 1995; Lively and Dybdahl 2000), parasites may impose negative frequency‐dependent selection that promotes host diversity, as in the Red Queen hypothesis (Lively 1987; Bérénos et al. 2011). In testing this second hypothesis, we determine if there is evidence for these scenarios and more broadly ask to what extent results from dynamic natural populations resemble those from constrained experimental host populations.

These untested hypotheses highlight the need for a systematic examination of the monoculture effect across the contexts in which it has been tested. Variation across contexts carries consequences for how we apply genetic diversity as a management tool in agriculture and conservation and for how we interpret and test foundational coevolutionary theory. To address this need, we quantified the relationship between intraspecific genetic diversity and parasitism over a diverse collection of studies. First, we tested for a reduction in parasitism with increased genetic diversity of experimental populations of noncrop and crop hosts in lab and field settings. Second, we measured the correlation between genetic diversity and parasitism in observational surveys of natural populations. Finally, we directly compared the results of these three study types: experiments in noncrop systems, experiments in crop systems, and observational surveys of noncrop populations. We find that diversity reduces parasitism in experimental populations, particularly of crop hosts, but we found no consistent relationship between diversity and parasitism in natural populations.

## Methods

We preregistered the protocol for this study (Gibson 2018).

### LITERATURE SEARCH

In September 2018, we searched PubMed and Web of Science for titles with the following terms: (“genetic diversity” OR “genetic variation” OR monoculture OR polyculture OR inbred OR inbreeding OR mixtur* OR heterogen* OR polyandr* OR polygyn* OR promisc* OR outcros*) AND (parasit* OR infect* OR pathogen OR disease OR susceptib* OR epidemic OR resist*)

**Figure 1:**
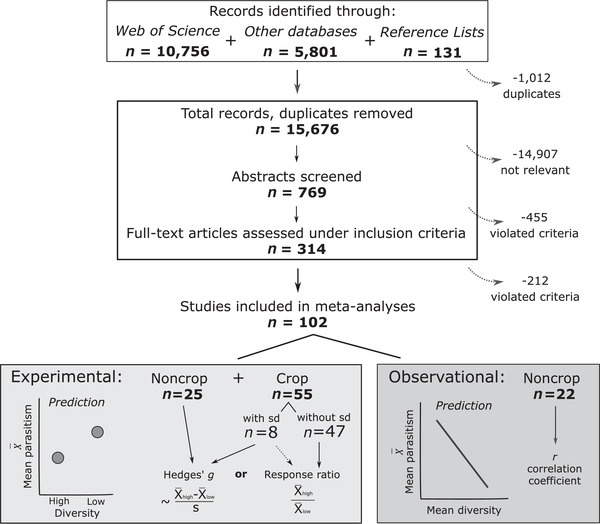
**Overview of literature search results and categorization of studies**. After literature searching and screening, the 102 studies that met our inclusion criteria were divided into experimental studies, where researchers controlled the genetic diversity and parasite exposure of experimental host populations, and observational studies, where researchers measured parasitism and genetic diversity across unmanipulated populations. Experimental studies were classified by host to distinguish noncrop from crop systems. Crop studies were further divided according to whether they provided an estimate of sampling variance (“sd” for standard deviation). We outline the predictions and effect sizes for these study types. We used the log response ratio (*lnRR*) and Fisher's*z*for statistical analyses (Table S2), but here and in the Results we present these effect sizes as the response ratio and correlation coefficient (*r*) because they are easier to interpret.

We received weekly Web of Science alerts of new search results through December 2019. We also searched Agricola (abstracts with genetic diversity AND disease resistance; monoculture AND pathogen) and ProQuest (abstracts with genetic diversity AND [disease resistance OR pathogen OR parasite]). Finally, we searched reference lists of reviews (e.g., King and Lively 2012; Mikaberidze et al. 2015), meta‐analyses, (e.g., Huang et al. 2012; Reiss and Drinkwater 2018; Ekroth et al. 2019), and all full studies examined for inclusion. Our search generated 15,676 unique records (Fig. 1).

### STUDY SELECTION

We evaluated full articles written in English reporting original data on variation in parasitism and host genetic diversity. We required population‐level data for multiple populations, natural or experimental. Studies were excluded if data were limited to a single population, individual‐level estimates (e.g., genomic heterozygosity), a subset of host genotypes, or transformed values. Genetic diversity of hosts had to be intraspecific and explicitly controlled or measured.

We use the term “parasite” to refer to parasites and pathogens (Lafferty and Kuris 2002). We rejected studies that only reported aggregate data for diverse parasites because this prevented a test of whether effect size varied with parasite traits. We accepted several quantitative estimates of population‐level parasitism, including prevalence (fraction of individuals scored as infected), mean load (a quantification of how infected hosts were), and virulence (parasite‐induced mortality). We did not accept metrics that were qualitative (i.e., presence/absence of parasites) or unlinked to transmission (i.e., immune responses). We did not consider general estimates of host fitness—these were rarely provided in eligible studies. Moreover, the association between diversity and mean fitness lies outside the scope of this study and has been addressed elsewhere (Reed and Frankham 2003; Leimu et al. 2006; Reiss and Drinkwater 2018).

We remained flexible in our calculation of effect sizes to include as many studies as possible (details to follow). Nonetheless, we excluded a few studies from which we could not calculate an appropriate effect size—one noncrop experimental study that did not report standard deviations and four observational studies with fewer than four sampled populations.

One hundred and two studies met our inclusion criteria (Fig. 1). We attribute the reduction in sample size to the fact that our search terms captured topics that were not relevant, such as the genetic diversity of parasites themselves or the relationship between parasitism and the genetics of individuals rather than populations (see Files S1 and S2 for details of excluded records). We present our approach to examining publication bias in the Supporting Information.

### DATA COLLECTION

Both authors extracted and checked all data. We collected data from tables, figures (WebPlotDigitizer 4.2: Rohatgi 2019), and supplements. We calculated different standardized effect sizes depending on study type and converted between them as needed (Borenstein et al. 2009a)—details are provided in the next section. To identify contextual factors that mediated the relationship between genetic diversity and parasitism, we collected data on biological and methodological variables. These variables are described in Table S1. We selected variables for analyses according to their relevance and sample size (details in subsequent sections) and to minimize their covariance.

### GENERAL APPROACH TO ANALYSES

We analyzed the data with multilevel models using the metafor package's rma.mv function in R version 3.6.0 (Viechtbauer 2010; R Core Team 2013). Some studies included multiple experiments or surveys, and some of these produced multiple effect sizes. There were also multiple effect sizes for some host genera (see Results in the Supporting Information). To account for this hierarchical structure, we specified random effects for host genus, study, and experiment/survey. When high‐diversity treatments shared the same low‐diversity (i.e., control) treatment, we followed Gleser and Olkin (2007) in estimating the sampling covariance of effect sizes or treated all effect sizes with shared controls as part of the same experiment (details in next section).

For each analysis, we first tested for the mean relationship between genetic diversity and parasitism. We then tested for variation among effect sizes using total *I* ^2^, which estimates the fraction of observed variation between effect sizes due to true heterogeneity rather than sampling variance (Higgins and Thompson 2002; Nakagawa and Santos 2012). *I* ^2^ values above 75% are interpreted as evidence of substantial heterogeneity, although Senior et al. (2016) estimated the median and mean *I* ^2^ to be 85% and 92%, respectively, for ecology and evolution meta‐analyses. If *I* ^2^ points to substantial heterogeneity, the strength of an effect may vary with context and a follow‐up analysis is recommended to identify factors that explain the variation (Nakagawa et al. 2017).

To identify contextual factors mediating the strength of the monoculture effect, we performed model selection analysis using the function dredge in the R package MuMIn (Barton 2019) to construct candidate models with all combinations of main effects among relevant biological and methodological factors. All but one factor was binary and we did not include interactions to avoid overfitting. Following Burnham and Anderson (1998), we compared candidate models using Akaike's information criterion corrected for small sample sizes (AIC_c_) and ΔAICc, the difference in AIC_c_ values of the focal model and the model with the lowest AIC_c_ (i.e., the best model). ΔAIC_c_ less than two generally indicates that a model has substantial support. We also calculated the Akaike weight (*w*), which is interpreted as the probability that a model is the best model among the candidates. If a clear set of best models (ΔAIC_c_ < ∼2; higher *w*) was identified, we further evaluated the factors in those models as potential contextual variables mediating the strength of the monoculture effect. Alternately, a large set of best models with low weights (*w* near 0) suggested that no single factor or subset of factors played a unique role in mediating the strength of the monoculture effect (Burnham and Anderson 1998).

We used conditional model averaging to determine the effect of contextual factors. We first identified the set of candidate models that contained the best model with 95% confidence, then estimated a factor's mean regression coefficient over all models in the set for which the factor was included (Burnham and Anderson 1998). This multimodel inference is recommended when there is uncertainty in identifying the best model because it provides a general estimate of a factor's effect across possible models. In light of debate over the value and interpretation of model‐averaged coefficients (Cade 2015; Banner and Higgs 2017; Walker 2019), we also fit models with each contextual factor individually.

### DETAILS OF ANALYSES BY STUDY TYPE

Studies reported the results of either experimental manipulations or observational surveys (Fig. 1). We defined experimental studies as those in which researchers controlled genetic diversity and parasite exposure of host populations. Researchers manipulated genetic diversity by varying the number of genotypes (inbred lines, clones, or cultivars), the number of males mated with foundress females, or the degree of inbreeding. They then standardized the exposure of experimental populations to parasites. We defined observational studies as those in which researchers played no role in manipulating genetic diversity or parasite exposure. These studies quantified the genetic diversity of field‐based populations using genetic markers and population genetic statistics, such as expected heterozygosity and diversity indexes.

We first conducted a separate meta‐analysis for each study type (Borenstein et al. 2009c) before conducting a combined analysis. Here, we provide details of each analysis.

#### Analysis of noncrop experiments

We calculated the relationship between genetic diversity and parasitism with the effect size Hedges’ *g*, the recommended statistic for experimental studies (Nakagawa et al. 2017). Hedges’ *g* gives the number of standard deviations by which mean parasitism differs between high‐ and low‐diversity host populations (see Table S2 for formulas). For three effect sizes in two studies (Giese and Hedrick 2003; Puurtinen et al. 2004), the experimental design required us to first calculate the correlation of parasitism with genetic diversity then convert to Hedges’ *g* (Borenstein et al. 2009a) (Table S2). In the model, we specified the sampling covariance for effect sizes calculated from shared low‐diversity controls (52 effect sizes across three studies). We examined 11 contextual variables: host taxon, parasite taxon, host reproductive mode, parasite virulence, parasite functional group, parasite host range, experimental setting, host population assembly, host diversity, parasitism metric, and experiment duration (Table S1).

#### Analysis of crop experiments

Eight crop studies fit our inclusion criteria and reported standard deviations, as required for calculating standard effect sizes for experimental studies. For these eight studies, we calculated Hedges’ *g*. These studies made extensive use of shared low‐diversity controls (89% of effect sizes), so we treated all effect sizes sharing controls as part of the same experiment. We examined four contextual variables: parasite life cycle, parasitism metric, mixture evenness, and inoculation mode (Table S1). In the next section, we describe an alternate approach to incorporate the 47 crop studies that did not report standard deviations.

#### Analysis of observation surveys

We calculated the relationship between genetic diversity and parasitism across populations with the effect size Fisher's *z*, the recommended statistic for correlational studies (Nakagawa et al. 2017). Fisher's *z* is derived from the sample correlation coefficient *r* (Table S2), and after analysis we transformed *z* back to *r* for interpretation (Borenstein et al. 2009b). We examined 10 contextual variables: host taxon, parasite taxon, host reproduction, parasite functional group, parasite host range, parasite life cycle, virulence, parasitism metric, conservation status (IUCN 2020), and island status (Table S1). Conservation and island status were added post hoc (Gibson 2018). We allowed candidate models to include at most four factors to limit overfitting.

#### Combined analysis of experimental and observation data

To further test observed differences between study types, we combined experimental and observational data by converting correlation coefficients to Hedges’ *g* (Table S2). In the model, we treated all effect sizes sharing low‐diversity controls as part of the same experiment. We included study type as a contextual factor. Experimental crop studies lacking standard deviations were excluded in this analysis because we were unable to convert to a common effect size. This exclusion did not undermine our goal for this analysis, which was to directly test for differences between study types. We did not aim to estimate the mean effect of genetic diversity on parasitism across all studies, because variation between study types suggested that this estimate was not meaningful.

### ALTERNATE ANALYSES FOR EXPERIMENTAL CROP STUDIES

Forty‐seven experimental crop studies did not report standard deviations. These represented the majority of the crop literature and spanned a wider time period (1977‐2017) than the eight studies with standard deviations (2000‐2019). To incorporate these studies, we applied an alternate effect size and weighting approach to all 55 studies. In contrast to Hedges’ *g*, the log response ratio (*lnRR*) can be calculated without standard deviations and weighted using sample sizes instead of sampling variances (Adams et al. 1997; Lajeunesse 2013) (Table S2). After analysis, we transformed *lnRR* back to the response ratio (ratio of mean parasitism in high‐ vs. low‐diversity treatments) for interpretation. We excluded 90 effect sizes because means of zero prevented calculation of *lnRR*.

To account for the hierarchical structure of the data, we calculated the weighted mean effect size for each experiment within a study and for each study. We then calculated the weighted means across experiments and studies. We calculated bootstrap confidence limits by sampling 10,000 times with replacement (Adams et al. 1997). To evaluate context dependence, we repeated this approach after splitting the dataset by two methodological factors, evenness and inoculation mode, and two taxonomic factors, membership in the Poaceae family and the genus *Triticum*. These taxonomic factors were not included in the smaller crop analysis due to insufficient sample sizes.

#### Comparable analyses of all experimental data

To compare all experimental studies, we repeated our prior analyses of studies with standard deviations using *lnRR* and its standard sampling variance (Lajeunesse 2011) (Table S2). Use of this standard sampling variance allowed us to apply the same models described previously, with a few modifications. We treated all effect sizes using shared controls as part of the same experiment. We excluded seven effect sizes from the noncrop dataset and one from the crop dataset because treatment means were zero or correlation coefficients could not be converted to *lnRR*. For the noncrop dataset, we also excluded an effect size whose small sampling variance prevented the model from running (Desai and Currie 2015) and the study Van Houte et al. (2016) because the scale of the data inflated response ratios. This was not an issue using Hedges’ *g*, which standardizes treatment differences by the scale of variation in the data. The results of the Hedges’ *g* analysis were unaltered by these exclusions, so the *lnRR* analysis served as a valid conversion.

## Results

We collected 2004 effect sizes from 102 studies (Fig. 1). These studies examined the monoculture effect in 45 host genera, 68 parasite genera, and 81 unique host‐parasite systems (Table 1). Experimental studies of noncrop systems showed the most taxonomic variety but focused on fungal parasites of invertebrate hosts, with 11 studies on social insects. Vertebrate hosts and animal parasites were more common in observational studies of noncrop systems. Plant hosts were rare in both types of noncrop studies. Crop experiments focused largely on fungal parasites of the major cereal grains—wheat (29 studies), oats, barley, and rice—although several noncereal crops were represented, including potatoes and apples. We found no evidence that our results were inflated by selective publication of small studies (Results in the Supporting Information).

**Table 1:** **Host and parasite genera represented in included studies**. Number of unique taxa, with the number of associated studies in parentheses

A. Host taxonomic distribution					
				*Plants*:	
	Bacteria	Invertebrates	Vertebrates	Poaceae	Other
*Experimental*					
Noncrop	3 (2)	9 (17)	3 (3)	0	3 (3)
Crop with sd^a^	0	0	0	3 (6)	2 (2)
Crop full^a^	0	0	0	4 (44)	7 (11)
*Observational*					
Noncrop	0	7 (10)	9 (9)	0	3 (3)
**Total unique**	3 (2)	14 (27)	12 (12)	4 (44)	12 (17)

B. Parasite taxonomic distribution^b^					
	Animals	Bacteria	Fungi^c^	Protozoa	Viruses
*Experimental*					
Noncrop	3 (3)	4 (5)	12 (16)	2 (3)	9 (5)
Crop with sd	0	0	5 (7)	0	1 (1)
Crop full	0	1 (1)	18 (52)	0	2 (3)
*Observational*					
Noncrop	9 (11)	4 (3)	2 (2)	5 (7)	3 (2)
**Total unique**	11 (14)	8 (9)	30 (70)	5 (10)	14 (10)

^a^ All experiments on crop plants were conducted using established cultivars. These cultivars are propagated via self‐fertilization, cloning (e.g., potato), or grafting (e.g., apple), minimizing genetic and phenotypic variation among individuals of the same cultivar.

^b^ Several studies reported data on multiple parasite genera.

^c^ Fungal and fungal‐like parasites, including microsporidia and oomycetes.

To evaluate the relationship between genetic diversity and parasitism, we first tested the prediction that genetic diversity reduces parasitism in experimental populations of noncrop and crop hosts. Second, we tested for a correlation of genetic diversity with parasitism in natural populations of noncrop hosts. Last, we directly tested for variation in the relationship of diversity and parasitism across study types.

### EXPERIMENTS IN NONCROP SYSTEMS

**Figure 2:**
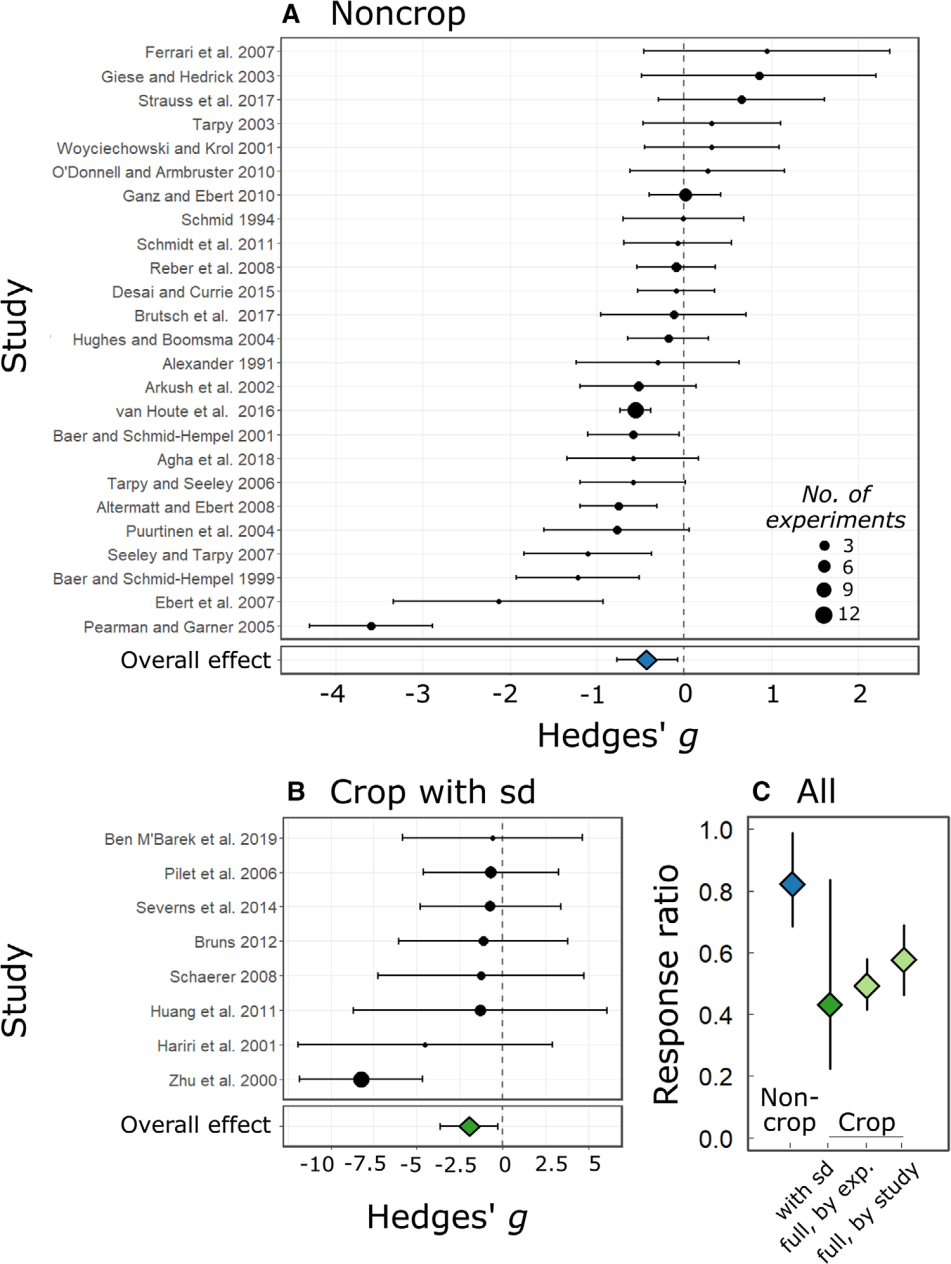
**Relationships between genetic diversity and parasitism in experimental studies**. Forest plots show the distribution of the standardized mean difference (Hedges’*g*) in parasitism between high‐ and low‐diversity host populations for 25 studies in noncrop systems (A) and eight studies in crop systems reporting standard deviations (sd) (B). Study‐level estimates and 95% confidence intervals were obtained from meta‐analytic models. The size of points is scaled by the number of independent experiments contributed by each study (min = 1, max = 12). Negative values of Hedges’*g*are consistent with less parasitism in diverse populations. Roughly, an estimate of |g|> 0.5 represents a large effect (treatment difference equal to or greater than ½ a standard deviation), >0.3 a moderate one, and >0.1 a small one. Note the difference in*x*‐axis scale between panels A and B. In panel C, the mean effect sizes across groups of experimental studies are compared using the response ratio, or level of parasitism in high‐diversity treatments divided by that in low‐diversity treatments. Smaller values of the response ratio are consistent with less parasitism in diverse populations, with an estimate of 0.5 indicating a reduction in parasitism of 50% with genetic diversity. The three different effect sizes for crop studies correspond to mean estimates for the subset of eight studies with standard deviations and the full set of crop studies analyzed either at the level of study or of independent experiment within study. Error bars show 95% confidence intervals obtained from meta‐analytic models (left two points) or by bootstrapping (right two points).

A negative effect of genetic diversity on parasitism would manifest as a negative standardized mean difference (Hedges’ *g*) between parasitism in high‐ and low‐diversity host populations (Fig. 1) (as in Civitello et al. 2015). In support of the monoculture effect, genetic diversity moderately reduced parasitism in experimental populations of noncrop hosts: *g* = −0.419 (95% CI [−0.766, −0.071], *P* = 0.018) (Fig. 2A). This estimate held under consideration of taxonomic biases (Results in the Supporting Information). *I* ^2^ was 82%, indicating substantial variation in the effect of genetic diversity on parasitism (Table 2).

**Table 2:** Summary of meta‐analysis results

Model	*N* ^a^	Studies	Experiments	Effect size	95% CI	SE	*z*	*P*‐value	*I* ^2^ ^b^
*Experimental*				*Hedges’ g*					
Noncrop	155	25	54	−0.419	[–0.766, −0.071]	0.177	−2.363	0.018	82.2
Crop with sd	225	8	34	−1.945	[−3.636, −0.254]	0.863	−2.254	0.024	91.5
				*Responseratio*					
Noncrop	83	24	38	0.822	[0.685, 0.987]	0.093	−2.098	0.036	
Crop with sd	224	8	34	0.431	[0.222, 0.835]	0.338	−2.494	0.013	
Crop full, by exp.	1769		235	0.493	[0.416, 0.579]				
Crop full, by study	1769	55		0.576	[0.462, 0.690]				
*Observational*				*Pearson's r*					
Noncrop	80	22	24	−0.029	[−0.210, 0.154]	0.094	−0.309	0.757	53.2
				*Hedges’ g*					
*Combined* ^c^	460	55	112	−0.570	[−0.873, −0.268]	0.154	−3.693	<0.001	84.5

^a^ *N* = number of effect sizes, followed by Studies = number of studies, and Experiments = number of total experiments or observational surveys. For the response ratio analysis of all crop studies, the overall effect is based upon 1769 effect sizes, summarized as 55 study‐level means or 235 experiment‐level means.

^b^ Total *I* ^2^ (%) estimates the total fraction of observed variation between effect sizes that is attributed to true heterogeneity between effect sizes, which combines heterogeneity among studies, experiments/surveys, and host genera.

^c^ This estimate excludes 47 crop studies that did not report standard deviations. Thus this estimate does not represent the mean effect across all studies in our dataset.

We tested if 11 contextual factors explained this variation. Model selection analysis identified nine models with ΔAIC_c_ < 2 and low weights (0.05 ≤ *w* ≤ 0.12). The best model contained no contextual factors (Table S3A). We estimated the mean effect of each contextual factor by averaging its regression coefficient across the 95% confidence set of models. No contextual factor had a substantial and consistent effect on the relationship between genetic diversity and parasitism (Table S3B). Additionally, in models fit with factors individually, the mean effect of genetic diversity did not differ significantly between levels of any factor (Fig. S1). These results indicate a limited role for the tested contextual factors in explaining variation in the effect of genetic diversity.

### EXPERIMENTS IN CROP SYSTEMS

We similarly tested for a negative effect of genetic diversity on parasitism in eight experimental studies of crops with standard deviations. In support of the monoculture effect, genetic diversity strongly reduced parasitism: *g* = −1.945 ([−3.636, −0.254], *P* = 0.024) (Fig. 2B).

The *I* ^2^ of 91.5% indicated substantial variation in the effect of genetic diversity (Table 2), so we tested if effect sizes varied with four contextual factors. Model selection analysis identified five models with ΔAIC_c_ < 2 and moderately low weights (0.10 ≤ *w* ≤ 0.23). Each of these models included mixture evenness as a factor (Table S4A). Averaging values of regression coefficients across models, we found that genetic diversity reduced parasitism more when cultivars were mixed unevenly (model‐averaged coefficient = −1.150 [−1.661, −0.638]) (Table S4B). A model with only evenness showed that diversity strongly reduced disease in even mixtures (*g =* −1.308 [−2.520, −0.096], *P* = 0.034) but even more so in uneven mixtures (*g =* −2.523 [−3.747, −1.299], *P* < 0.001) (Fig. S2A). Diversity also reduced parasitism more when plants were directly inoculated with parasites (model‐averaged coefficient = −1.080 [−1952, −0.208]). A model with only inoculation mode showed that diversity reduced parasitism with direct inoculation (*g =* −2.425 [−4.036, −0.815], *P* = 0.003) but only marginally with passive parasite exposure in field settings (*g =* −1.369 [−2.971, 0.234], *P* = 0.094) (Fig. S2B).

The alternate analysis of all 55 crop studies further supported a major reduction in parasitism with genetic diversity. In this analysis, a negative effect of genetic diversity on parasitism would manifest as a value below one for the response ratio (i.e., mean parasitism in mixtures divided by that in monocultures). The weighted mean response ratio across the 235 experiments was 0.493 (bootstrapped 95% CI [0.416, 0.579]), indicating a reduction in parasitism of over 50% in mixtures relative to monocultures. The weighted mean response ratio across the 55 studies was consistent with this result (0.576 [0.462, 0.690]) (Table 2). To follow up on prior findings of context dependence, we estimated the weighted mean response ratio for levels of different contextual factors. The results supported variation in effect sizes with mixture evenness but not inoculation mode. We also found a stronger effect of genetic diversity for cereal crops (Poaceae) than for noncereal crops (Table S5).

In comparing all experimental datasets via response ratios, we found that the subset of eight crop studies reporting standard deviations showed a similar effect as the full crop dataset: the mean response ratio for the subset was 0.431 [0.222, 0.835] (Table 2). Genetic diversity had a weaker effect on parasitism in noncrop experiments: the mean response ratio was 0.822 [0.685, 0.987], indicating a reduction in parasitism of ∼20% with increased genetic diversity (Fig. 2C).

### OBSERVATIONAL SURVEYS OF NONCROP SYSTEMS

**Figure 3:**
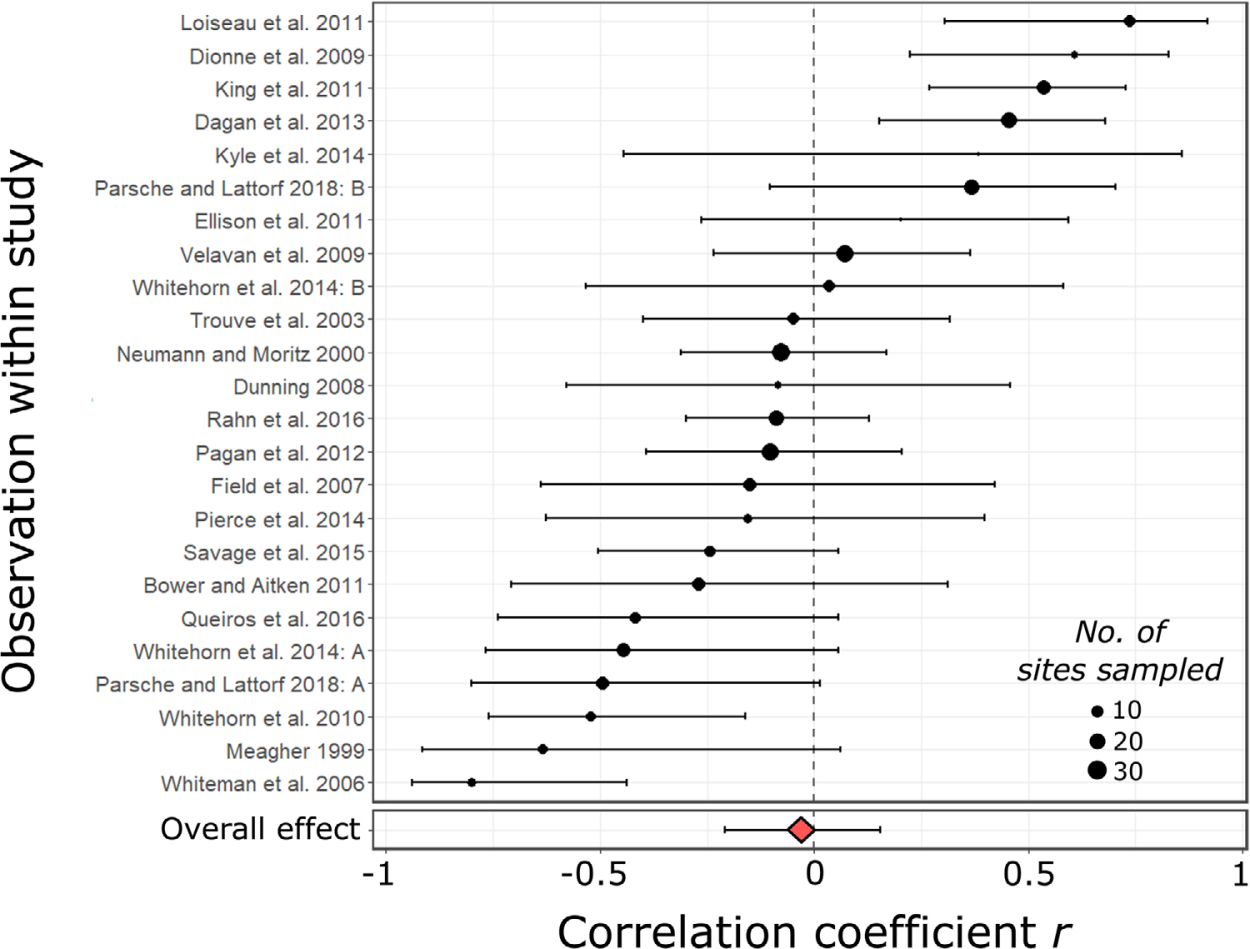
**Relationship between genetic diversity and parasitism in observational studies**. Forest plots show the distribution of the correlation coefficient*r*relating population‐level estimates of parasitism and genetic diversity across field‐based populations of noncrop hosts. Estimates are shown for 24 observational surveys in 22 studies—Parsche and Lattorf (2018) and Whitehorn et al. (2014) reported two independent surveys with data from two different host species. Observation‐level estimates and 95% confidence intervals were obtained from a meta‐analytic model. The size of points is scaled by the number of sampled sites included in each survey (min = 4, max = 30).

Following on these experimental results, we tested for a negative correlation between parasitism and population genetic estimates of genetic diversity across natural host populations (Fig. 1). There was no support for a negative relationship: the average correlation coefficient was indistinguishable from zero (*r* = −0.029 [−0.210, 0.154], *P* = 0.757) (Table 2) (Fig. 3). This finding held after excluding studies with some managed populations (Results in the Supporting Information).

The *I* ^2^ of 53.2% indicated moderate variation in the effect of genetic diversity (Table 2), so we tested if effect sizes varied with 10 contextual factors. Model selection analysis identified five models with ΔAIC_c_ < 2 and low weights (0.03 ≤ *w* ≤ 0.07). All top models included conservation status and parasite life cycle (Table S6A). Averaging values of the regression coefficients across models, we found that the correlation between diversity and parasitism varied substantially with conservation status (model‐averaged coefficient, threatened vs. not = 0.505 [0.118, 0.759]), parasite life cycle (simple vs. complex = 0.267 [0.002, 0.498]), and island status (island vs. mainland = 0.442 [−0.014, 0.746]) (Table S6B). Fitting models with factors individually, we found a negative correlation of genetic diversity with parasitism for threatened taxa (*r =* −0.535 [−0.752, −0.213], *P* = 0.002) but not unthreatened taxa (0.082 [−0.077, 0.236], *P* = 0.313). This distinction weakened when we relaxed our criterion for classification as threatened (Results in the Supporting Information). We also found a negative correlation across island (*r =* −0.457 [−0.676, −0.164], *P* = 0.003) but not mainland populations (*r* = 0.102 [−0.640, 0.263], *P* = 0.227). Finally, a model with only parasite life cycle found a negative correlation for simple life cycle parasites (*r =* −0.138 [−0.310, 0.044], *P* = 0.137) and a positive one for complex life cycle parasites (*r =* 0.182 [−0.048, 0.393], *P* = 0.120), but these did not differ significantly from zero (Fig. S3).

### COMBINED ANALYSIS OF EXPERIMENTAL AND OBSERVATIONAL DATA

**Figure 4:**
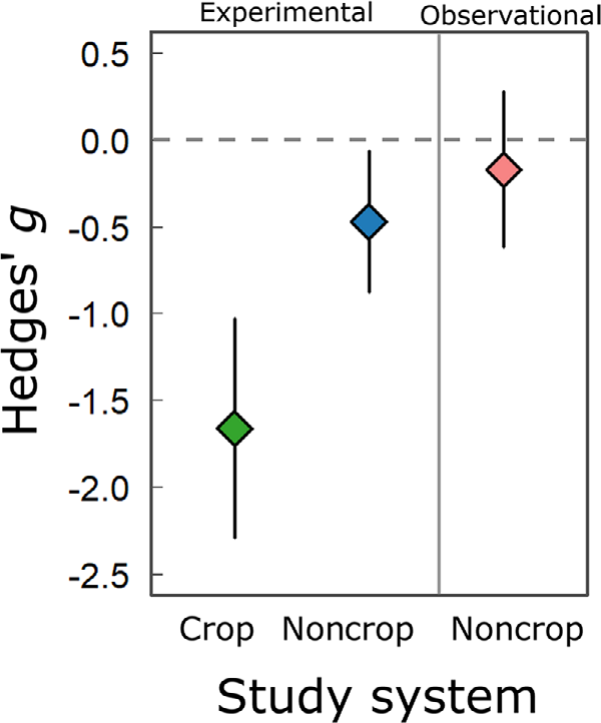
**Relationship between genetic diversity and parasitism across experimental and observational studies**. Average Hedges’*g*estimates are given for the eight experimental crop studies reporting standard deviations, all experimental studies in noncrop systems, and all observational studies of natural populations. Subgroup estimates and 95% confidence intervals were obtained from a meta‐analytic model. Negative values of Hedges’*g*are consistent with less parasitism in diverse populations.

Finally, we directly tested whether study type contributed to variation among effect sizes in a combined dataset. Across experimental and observational data, genetic diversity had a negative relationship with parasitism (*g =* −0.570 [−0.873, −0.268], *P* < 0.001) with an *I* ^2^ of 84.5% (Table 2). Including study type as a contextual factor, we found that genetic diversity reduced parasitism most strongly for crops: the effect was far more negative in experimental populations of crops than noncrop hosts (coefficient = −1.189 [−1.194, −0.438], *P* = 0.002) (Fig. 4). We also found that surveys of natural host populations detected a weaker relationship between genetic diversity and parasitism than tests in experimental host populations: experimental studies of noncrop hosts again showed a significant negative relationship between genetic diversity and parasitism (*g =* −0.472 [−0.878, −0.067], *P* = 0.023), while observational studies did not (*g =* −0.169 [−0.617, 0.278], *P* = 0.458) (Table S7), although the mean estimates for these two groups overlapped (coefficient = 0.303 [−0.301, 0.907], *P* = 0.325).

## Discussion

We set out to test the hypothesis that genetic diversity limits parasitism. By conducting a series of meta‐analyses representing the largest, most comprehensive analysis of this hypothesis to date (Fig. 1), we show extensive variation in the effect of genetic diversity on parasitism. On average, genetic diversity reduced parasitism in experimental host populations (Fig. 2), particularly for crops: the mean effect size was approximately threefold greater for crops than for noncrop hosts (Fig. 2C). In contrast, we found no mean correlation between genetic diversity and parasitism in natural host populations (Fig. 3). These findings support our hypotheses predicting variation across study types. Taken together, this work establishes the monoculture effect as a broadly relevant phenomenon, the estimated strength of which varies across biological and empirical contexts (Table 2; Fig. 4).

Table 1 attests to the phylogenetic breadth of the literature on the monoculture effect: it has been tested in 81 unique host‐parasite combinations. Only three host genera, five parasite genera, and one host‐parasite system were represented in more than one type of study. For experimental studies, this lack of overlap indicates that genetic diversity's protective effect holds across a diversity of hosts. Consistent with this, effect sizes varied little between broad taxonomic groupings of hosts and parasites (Results in the Supporting Information; Figs. S1 and S3).

Nonetheless, the lack of overlap between study types points to significant taxonomic gaps in the literature. Plant hosts were rare outside of crop studies, and experimental tests in noncrop hosts are biased toward invertebrates, with heavy representation of social insects and *Daphnia* hosts (Table 1). Thus there may be meaningful variation in the effect of genetic variation across host taxonomic groups that we have limited power to detect in the current body of literature. Moreover, the variation in results across study types may stem in part from their taxonomic divergence. Notably, we cannot rule out a role for plants in the extreme monoculture effect for crops. The three experimental studies on noncrop plants did not report large, negative effects (Alexander 1991; Schmid 1994; Ferrari et al. 2007) (Fig. 2A), but this sample is insufficient for comparison. It is also exceedingly rare for the monoculture effect to be tested in natural and experimental populations of the same host‐parasite system (although see Neumann and Moritz 2000; Desai and Currie 2015). Such pairings are necessary to test hypotheses for the divergent results in experimental versus observational studies.

### EXPERIMENTS IN NONCROP SYSTEMS

For noncrop experiments, we estimated an effect (*g* = −0.419) similar to that of Ekroth et al. (2019) (converted *g =* −0.353) (Fig. 2A). Reversal of effect size signs from Ekroth et al. (2019) allows direct comparison with our results. Our two datasets were not identical: they shared 14 experimental studies, but we included 11 additional studies and excluded six from Ekroth et al. (2019) that did not meet our criteria. In addition, Ekroth et al. (2019) included three studies that we classified as observational (File S2). The correspondence of our mean effects with semi‐independent datasets speaks to the robustness of the moderate reduction in parasitism with increased diversity of noncrop hosts.

Both Ekroth et al. (2019) and our study found substantial variation in the effect of genetic diversity. Ekroth et al. (2019) linked this variation to study setting and parasite functional group: genetic diversity reduced parasitism in field settings but not lab settings and for microparasites but not macroparasites. In contrast, we did not identify any contextual factors that explained variation in effect sizes (Table S3; Fig. S1). This may reflect differences in our datasets. Notably, Ekroth et al. (2019) included three field‐based observational studies reporting large negative effects. Our results suggest that setting and functional group do not broadly explain variation in the effect of genetic diversity. Because the wide range of approaches in experimental studies may limit the power of meta‐analyses, we advocate for direct tests of contextual factors for which we have *a priori* hypotheses. Modeling this approach, Ganz and Ebert (2010) found that host diversity limited parasitism only when the parasite population itself was genetically diverse. We could not analyze parasite diversity as a contextual factor because it covaried with experimental setting (Table S1). Replications of Ganz and Ebert (2010) would allow tests of parasite diversity as a general driver of variation in the effect of host diversity.

### COMPARISON WITH CROP EXPERIMENTS

Genetic diversity had the greatest effect in experimental populations of crops. On average, mixtures of two or more cultivars experienced half the parasitism of monocultures (Figs. 2B and 2C). This is nearly double the mean effect estimated by the only other meta‐analysis of genetic diversity and parasitism in crops. Huang et al. (2012) focused on stripe rust of wheat, whereas we synthesized data from 30 crop‐parasite systems. Strikingly, the effect of crop genetic diversity on parasitism is nearly double the effect of host species diversity (Civitello et al. 2015).

We hypothesize that this massive effect reflects the fact that host resistance phenotypes were known in 54 of 55 crop studies (exception: Bruns 2012). Crop mixtures had both genetic diversity *and* phenotypic diversity. In contrast, in 22 of 25 noncrop studies, researchers assembled mixtures blind to resistance phenotypes (exceptions: Schmid 1994; Ebert et al. 2007; Strauss et al. 2017). The greater effect in uneven relative to even cultivar mixtures supports this argument (Table S4; Fig. S2): >50% of uneven mixtures were biased toward resistant cultivars, magnifying reductions in parasitism in mixtures. Similarly, the stronger effect in cereal crops (Table S5) may stem from there being more cereal cultivars with greater resistance variation than for fruit and vegetable crops (Mundt 2002; European Commission 2019).

These unique elements of experimental design and host genetics advise caution in extrapolating beyond crops. The extreme effect in crop experiments is a best case scenario, in which genetic diversity directly translates to resistance diversity (Hughes et al. 2008). Nonetheless, declining host populations may approach the genetic impoverishment of crop monocultures. Indeed, for noncrop hosts the largest reduction in parasitism with genetic diversity came from experimental populations that replicated the loss of genetic diversity in vulnerable versus stable populations of the Italian agile frog *Rana latastei* (Pearman and Garner 2005) (Fig. 2A).

### COMPARISON WITH OBSERVATIONS OF NATURAL POPULATIONS

In contrast to experimental populations, we found no mean correlation between genetic diversity and parasitism across natural host populations (Fig. 3). The effect of genetic diversity may simply be weaker in field settings. However, we did not detect a difference between lab and field‐based experiments (Table S3; Fig. S1D), and Ekroth et al. (2019) reported a stronger effect of genetic diversity in field studies. Moreover, we did not detect predominantly weak effect sizes for observational surveys. Figure 3 shows both strong negative and positive correlations of genetic diversity with parasitism. This distribution contrasts with experimental studies, where effect sizes were rarely positive (Fig. 2A).

Wide variation in the correlation coincides with the hypothesis that the same mechanism—the reduced probability that genetically dissimilar hosts contract the same infection—can generate negative or positive relationships between genetic diversity and parasitism. A negative correlation would arise when host populations do not evolve in response to parasite selection, akin to the relative stasis of experimental host populations. Meagher (1999) argued that he found a negative correlation between nematode parasitism and genetic diversity because founder events dictated the genetic structure of deer mice populations on Michigan islands. We similarly found negative correlations for host populations distributed across islands (Table S6; Fig. S3). We also found a negative correlation for threatened hosts, for whom external forces may degrade genetic diversity and prevent rapid adaptation. The general significance of threatened status is unclear because our dataset only included three threatened species (see Results in the Supporting Information). Nonetheless, this result bolsters the importance of conserving genetic diversity to buffer threatened taxa against epidemics (O'Brien and Evermann 1988).

A positive correlation between genetic diversity and parasitism could arise if host populations evolve in response to parasite selection. Tests of the Red Queen hypothesis support parasites as agents of negative frequency‐dependent selection that maintain diversity in coevolving host populations (e.g., Schmitt and Antonovics 1986; Morran et al. 2011). Our results provide weak support for an association of positive correlations with complex life cycle parasites. This association may stem from the fact that two of the strongest positive correlations come from snail‐trematode systems (complex life cycle parasites) (King et al. 2011; Dagan et al. 2013) (Fig. 3) for which there is evidence of parasite‐mediated negative frequency‐dependent selection (Koskella and Lively 2009).

Ultimately, our results leave us with the question: in general, for freely evolving host populations, when do we expect parasites to track host diversity, and when do we expect them to promote host diversity by preferentially attacking common lineages? Future work should pair tests in experimental host populations with observational surveys to determine how an underlying protective effect of genetic diversity manifests in dynamic natural populations.

## Conclusion

Our study validates a hypothesis that has long been accepted at face value (King and Lively 2012). In agriculture, speculation on disease in monocultures traces back to at least the late 1700s (Tozzetti 1767). Our results establish the enormous potential of cultivar diversification as a sustainable solution for disease control and yield improvement (Kiær et al. 2009; Reiss and Drinkwater 2018). In evolutionary ecology, parasitism emerged as an explanation for the advantage of strategies, such as sex and polyandry, that can maintain genetic diversity (Haldane 1949). Our results support the underlying assumption of these ideas: genetic diversity substantially reduces parasitism in experimental host populations. We also highlight opportunities for expanding the taxonomic scope of these experimental tests. Finally, although the relationship between genetic diversity and parasitism varies widely in natural populations, our results underscore the threat posed by epidemics if external forces turn wild populations into monocultures.

## AUTHOR CONTRIBUTIONS

AKG conceived and directed the study, conducted the literature search, study selection, data extraction, and data analysis, and wrote the manuscript. AN helped guide the study, conducted the study selection and data extraction, contributed to data analysis, and critically revised the manuscript.

## DATA ARCHIVING

Literature search records (S1), inclusion evaluations (S2), extracted data, calculations of effect sizes (S3), and analysis scripts (S4‐S8) are available as Supporting Information for review on the GitHub Repository genetic‐diversity‐and‐disease. Data files and analysis scripts are also deposited in the Dryad Digital Repository https://doi.org/10.5061/dryad.dv41ns1wg. An Endnote file containing the full list of titles from the database searches is available from the authors upon request.

Associate Editor: R. Snook

## Supporting information

Supplementary Material

**Table S1**. Biological and methodological variables included in tests for context dependence.
**Table S2**. Formulas used in effect size calculations
**Table S3**. Analysis of context dependence in experimental studies of noncrop systems.
**Table S4**. Analysis of context dependence in the subset of crop studies reporting standard deviations.
**Table S5**. Analysis of context dependence in the full collection of crop studies.
**Table S6**. Analysis of context dependence in observational studies of noncrop systems.
**Table S7**. Combined analysis of experimental and observational data.
**Figure S1**. Testing for context dependence in experimental studies of noncrop systems.
**Figure S2**. Testing for context dependence in experimental studies of crop systems.
**Figure S3**. Testing for context dependence in observational studies of noncrop systems.

Supplementary Material

Supplementary Material
