# Supplementary material for: Does genetic diversity protect host populations from parasites? A meta‐analysis across natural and agricultural systems
Source: Evol Lett. 2020 Nov 14;5(1):16–32. doi: 10.1002/evl3.206 (PMC7857278; doi:10.1002/evl3.206)
Supplement: Supplementary file 2 — Table S1. Biological and methodological variables included in tests for context dependence. Table S2. Formulas used in effect size calculations Table S3. Analysis of context dependence in experimental studies of noncrop systems. Table S4. Analysis of context dependence in the subset of crop studies reporting standard deviations. Table S5. Analysis of context dependence in the full collection of crop studies. Table S6. Analysis of context dependence in observational studies of noncrop systems. Table S7. Combined analysis of experimental and observational data. Figure S1. Testing for context dependence in experimental studies of noncrop systems. Figure S2. Testing for context dependence in experimental studies of crop systems. Figure S3. Testing for context dependence in observational studies of noncrop systems. [file EVL3-5-16-s002.docx]

**SUPPLEMENT**

Supplemental Results

**Publication Bias**

For each of our meta-analyses, there was non-independence among effect sizes and extensive heterogeneity. Non-independence and heterogeneity invalidate standard tests for publication bias, like funnel plot asymmetry tests. Though modified tests for funnel plot asymmetry have been suggested for multilevel models (Nakagawa and Santos 2012), many factors other than publication bias may cause funnel plot asymmetry even with standard datasets (Lau et al. 2006; Sterne et al. 2011). Thus the results of these tests are at best difficult to interpret and at worst misleading. We followed the recommendation of Sterne (2011) for datasets with between-study heterogeneity. Tests of publication bias assume that studies with small sample sizes (hence, large sampling variance) and small to moderate effect sizes are the most likely to go unpublished. The concern then is that preferential publication of small studies with large effect sizes biases the analysis. To determine if smaller studies biased our results, we compared the results of fixed and random effects analyses based on study-level means and sampling variances. Random effects analyses give more weight to smaller studies than fixed effects analyses, so substantial differences in the estimates from fixed and random effects analyses could potentially indicate bias induced by small studies. We obtained study-level means and sampling variances from multilevel models specifying study as a factor.

*Experiments in non-crop systems*: We found no evidence of bias due to selective publication of small studies: random and fixed-effects analysis of study-level means produced similar estimates of Hedges' *g* (random = -0.420 [-0.767, -0.072], p<0.018; fixed = -0.458 [-0.563, -0.354], p<0.001).

*Experiments in crop systems*: For the subset of studies with standard deviations reported, comparison of random and fixed-effects analyses of study-level means indicated that this estimate was not inflated by selective publication of small studies (random, *g* = -1.872 [-3.209,-0.535], p=0.006]; fixed, *g* = -1.870 [-3.021, -0.719], p=0.002). We further pursued the potential for publication bias by conducting an additional, alternative analysis that incorporated a much larger body of the crop literature (see Main Text).

*Observational surveys of non-crop systems*: Because relatively few studies reported multiple observational surveys, we obtained means and sampling variances at the level of survey rather than study. This analysis accordingly asks if observations based on a smaller number of natural populations biased our results. We found no evidence of this: the results from random and fixed-effects analyses were similar (random, *r* = -0.039 [-0.214,0.138], p=0.668]; fixed, *r* = -0.031 [-0.169, 0.108], p=0.664).

**Further considerations of taxonomic biases**

Several studies tested the relationship between genetic diversity and parasitism on the same host species or genus. To account for the non-independence of these effect sizes, we specified a random effect for host genus in all analyses (similar to Civitello et al. 2015; Ekroth et al. 2019), which grouped effect sizes collected from host species in the same genus (e.g. those from *Daphnia magna* and *D. dentifera* in experimental studies of non-crop hosts). We also repeated analyses with a nested taxonomic random effect, with genus nested within the highest taxonomic grouping with at least five levels (as in Hargreaves et al. 2020). This highest taxonomic grouping was phylum for both observational and experimental studies of non-crop hosts. Inclusion of this nested random effect did not alter our results for either non-crop analysis or the combined analysis, so we report the results of analyses with just the host genus random effect in the main text. For the subset of eight crop studies, the highest taxonomic grouping with five levels was genus, so we did not include a nested taxonomic random effect for this analysis. We did not include a formal phylogenetic correction in our analyses because the extreme taxonomic breadth and distribution of included host species (Table 1) raised the problem that long branches could bias phylogenetic analyses (as in Hargreaves et al. 2020; Uyeda et al. 2018).

*Experiments in non-crop systems*

Here, we further examine the significance of taxonomic biases specific to experimental studies of non-crop hosts. First, we repeated the analysis without data from the European honeybee *Apis mellifera*, which is at least partially domesticated (Oxley and Oldroyd 2010). Excluding five studies on *A. mellifera* did not change the mean effect of genetic diversity on parasitism in experimental studies of non-crop hosts (*g =* -0.458 [-0.882, -0.035], p=0.034).

Second, we tested if the relationship between genetic diversity and parasitism was stronger for social insects, which were common among experimental studies of non-crop hosts (47 effect sizes). Many tests of the monoculture effect have been conducted in social Hymenoptera because the idea served as an important hypothesis for polyandry in social insects (Sherman et al. 1988). The high density and low genetic diversity associated with colonies of many social insect taxa suggest that the monoculture effect may act relatively strongly for these groups, akin to crop plant hosts. We note, however, that four of the six social insect taxa included in our dataset show behaviors, like polyandry or polygyny, that can increase genetic diversity within colonies (*Apis mellifera, Acromyrmex echinatior, Formica selysi, Lasius niger* - Brütsch et al. 2017; Desai and Currie 2015; Hughes et al. 2008; Hughes and Boomsma 2004; Reber et al. 2008). Moreover, we detected no consistent differences in experimental design between experiments on social insects vs. other taxa.

We nonetheless tested if social insects differed systematically from other host taxa. We could not include social insect status in the contextual analysis in the main text because it covaried with a broader taxonomic distinction between invertebrate and non-invertebrate hosts. Instead, we conducted an analysis with social insect status as the sole contextual factor. We found no evidence that the effect of genetic diversity on parasitism differed for social insects relative to other hosts in the dataset (coefficient = 0.201 [-0.506, 0.908], p=0.578), though power to detect such a difference could be limited by sample size.

**Additional contextual analysis for observational surveys of non-crop systems**

First, we repeated the analysis of observational studies excluding data from host populations subject to any management by humans. Our observational dataset included one study on *A. mellifera* (Neumann and Moritz 2000), as well as two studies that included both natural and managed host populations (wild pepper *Capsicum annuum* var. *glabriusculum*: Pagán et al. 2012; red deer *Cervus elaphus*: Queirós et al. 2016). Though these studies cannot be considered to be investigations of purely natural populations, we included them in the observational dataset because they addressed our focal question and researchers did not manipulate parasite exposure or genetic diversity. The mean correlation of genetic diversity and parasitism was unaffected by exclusion of these studies (*r =* 0.012 [-0.207, 0.229], p=0.918). Management of host populations should limit their potential to respond to parasite selection. Hence, the argument outlined in the main text predicts a negative correlation between genetic diversity and parasitism across managed host populations. Consistent with this prediction, these studies all reported negative mean correlations (Fig. 3).

Second, we repeated our analysis of contextual factors relaxing our criterion for classifying taxa as threatened. In the main text, we report an analysis in which taxa are classified as threatened if they are listed by the IUCN Red List (2020) as vulnerable or endangered globally or in the region in which the study was conducted (Table S1). Based on this independent categorization, three host species across four studies were classified as threatened. Two studies reported population declines for their focal host species (*Salmo salar*: Dionne et al. 2009; *Lithobates yavapaiensis*: Savage et al. 2015), but both species are listed as “Least Concern” with the IUCN Red List (see also COSEWIC 2010 for *S. salar*). We repeated the analysis categorizing these two host species as threatened. With this less stringent categorization, threatened status no longer appeared as an important contextual factor in the top models. In its stead, island status increased in importance, appearing in all the top models with a larger model-averaged coefficient (island vs. mainland = 0.531 [0.213, 0.747]) (vs. Table S6). In a model fit with this new threatened status as the sole contextual factor, we still found a negative correlation of parasitism and genetic diversity for threatened taxa (*r =* -0.236 [-0.534,0.114], p=0.184) and none for unthreatened taxa (*r =* 0.073 [-0.134,0.275], p=0.491), but these correlations were no longer significant (vs. Fig. S3). This change likely reflects the very strong positive correlations between genetic diversity of *S. salar* and *Myxobolus* parasites reported in Dionne et al. (2009) (Fig. 3).

Supplemental Tables

| **Table S1: Biological and methodological variables included in tests for context-dependence**. Supplemental raw data files include further information on how we categorized data. Unless otherwise noted, if a variable was not considered for an analysis, it is because it was either not relevant or sample sizes were limited in one or more of the categories. |
| --- |
| \| **Biological** \| **Description** \| **Included in analysis of:** \| \| --- \| --- \| --- \| \| Host taxon \| Invertebrate vs. Other;  Social insect vs. Other;  Poaceae vs. Other;  *Triticum* vs. Other; \| Experimental non-crop + Observational;  Experimental non-crop - Supplemental Results;  Experimental crop – full;  Experimental crop – full \| \| Parasite taxon \| Fungal vs. Other; \| Experimental non-crop; \| \|  \| Animal vs. Other \| Observational \| \| Host reproduction \| Obligate outcrossing vs. Selfing/asexual/mixed mating \| Experimental non-crop + Observational \| \| Virulence \| High (lethal/sterilizing) vs. Low \| Experimental non-crop + Observational \| \| Functional group \| Microparasite vs. macroparasite \| Experimental non-crop + Observational \| \| Host range \| Specialist (single host genus) vs. Generalist (broader) \| Experimental non-crop + Observational \| \| Life cycle \| Simple vs. Complex (multihost) \| Experimental crop – sd only + Observational \| \| IUCN status \| IUCN vulnerable or endangered vs. Least Concern \| Observational (post hoc) \| \| Island status \| Island vs. mainland populations \| Observational (post hoc) \| \|  \|  \|  \| \| **Methodological** \|  \|  \| \| Experimental setting \| Lab vs. Field \| Experimental non-crop \| \| Host population assembly \| Mating/inbreeding vs. Combining genotypes \| Experimental non-crop \| \| Host diversity in diverse treatments \| Relatively high (more than 10 genotypes/mates; mating of natural populations) vs. Low (fewer than 10 genotypes/mates; mating of inbred populations) \| Experimental non-crop \| \| Parasitism metric \| Prevalence (binary scoring of individuals as healthy or infected) vs. Load (quantitative assessment of how infected a host was) vs. Virulence (disease-induced mortality); \| Experimental non-crop \| \|  \| Prevalence vs. Load; \| Experimental crop – sd; \| \|  \| Prevalence vs. Load \| Observational (two effect sizes with virulence excluded) \| \| Experiment duration \| Short (~two or fewer generations of parasite spread) vs. Longer \| Experimental non-crop \| \| Evenness \| Even (genotypes represented equally in mixtures) vs. Uneven \| Experimental crop \| \| Inoculation mode \| Direct (artificial) vs. Passive (natural) \| Experimental crop;  excluded for experimental non-crop due to covariance with experimental setting \| \| Parasite diversity in inocula \| High (more than 10 genotypes or a sample from natural populations) vs. Low (fewer than 10 genotypes in an artificial mixture) \| Relevant to all experimental studies but excluded due to covariance with experimental setting and inoculation mode \| \| Nature of associated loci \| Neutral vs. immune-related (e.g. sequencing of MHC loci) \| Relevant for observational studies but excluded because immune-related loci were rare \| |

| **Table S2: Formulas used in effect size calculations** |
| --- |
| \| **Effect size statistic** \| **Formula** \|  \| \| --- \| --- \| --- \| \| Hedges' *g* \| $g=\frac{\bar{X}_{high}-\bar{X}_{low}}{s}J$ \| Difference between mean parasitism ($\bar{X)}$ in experimental host populations with high vs. low diversity, standardized by the within-groups standard deviation *s* and multiplied by sample size correction factor *J* \| \|  \| $s=\sqrt{\frac{\left( n_{high}-1 \right)S_{high}^{2}+\left( n_{low}-1 \right)S_{low}^{2}}{n_{high}+n_{low}-2}}$ \| *n* indicates the number of experimental replicates per treatment; *S* the group’s standard deviation \| \|  \| $J=1-\frac{3}{4df-1}$ \| Sample size correction factor, *df* = degrees of freedom (*n_high+_n_low_*-2, for two treatment groups) \| \|  \| $V_{g}=J^{2}(\frac{n_{high}+n_{low}}{n_{high}n_{low}}+\frac{\left( \frac{\bar{X}_{high}-\bar{X}_{low}}{s} \right)^{2}}{2*(n_{high}+n_{low})})$ \| Sampling variance of *g* \| \|  \| $V_{g}=J^{2}(\frac{n_{high}+n_{low}}{n_{high}n_{low}}+\frac{\left( \frac{\bar{X}_{high}-\bar{X}_{low}}{s} \right)^{2}}{2*n_{total}})$ \| Variance of *g* when multiple experimental treatments share a low diversity control treatment, where *n_total_* is the total sample size among high diversity treatments and the shared low diversity treatment \| \|  \| ${cov}_{g}=\frac{1}{n_{low}}+\frac{g_{high1}g_{high2}}{2*n_{total}}$ \| Covariance between high diversity treatments with a shared low diversity treatment \| \| log response ratio *lnRR* \| $lnRR=ln\left( \frac{\bar{X}_{high}}{\bar{X}_{low}} \right)$ \| Log response ratio was used as the effect size in meta-analyses and converted to the response ratio (X_high_/X_low_) for presentation in the Results \| \|  \| $V_{RR}= \frac{S_{high}^{2}}{n_{high}\bar{X}_{high}^{2}}+\frac{S_{low}^{2}}{n_{low}\bar{X}_{low}^{2}}$ \| Standard sampling variance of lnRR \| \|  \| $W=\frac{n_{high}n_{low}}{n_{high}+n_{low}}$ \| Sample size weight for use if studies lack standard deviations \| \| Fisher's *z* \| $z=\frac{1}{2}ln\left( \frac{1+r}{1-r} \right)$ \| Fisher’s *z* was used as the effect size in meta-analysis and transformed back to the correlation coefficient *r* for presentation in the Results \| \|  \| $V_{z}=\frac{1}{n-3}$ \| Variance of *z,* where *n* is the number of populations sampled \| \| Conversions \| $g= \frac{2r}{\sqrt{1-r^{2}}}J$ \| Conversion of *r* to *g* \| \|  \| $V_{r}=\frac{\left( 1-r^{2} \right)^{2}}{n-1}$ \| Sampling variance of *r*. *n* is the number of populations sampled \| \|  \| $V_{g}=\frac{4*V_{r}}{\left( 1-r^{2} \right)^{3}}J^{2}$ \| Conversion to sampling variance of *g* \| |

| **Table S3: Analysis of context dependence in experimental studies of non-crop systems.** |
| --- |
| **A. Results of model selection analysis: top 20 models**   \| **Rank** \| **Terms** \| **N Factors** \| **AICc** \| **ΔAIC** \| **Weight *w*** \| \| --- \| --- \| --- \| --- \| --- \| --- \| \| 1 \| (intercept) \| 0 \| 376.7 \| 0 \| 0.120 \| \| 2 \| Functional group \| 1 \| 377.7 \| 0.97 \| 0.074 \| \| 3 \| Assembly \| 1 \| 377.8 \| 1.1 \| 0.069 \| \| 4 \| Parasite taxon \| 1 \| 378.1 \| 1.37 \| 0.060 \| \| 5 \| Host diversity \| 1 \| 378.2 \| 1.45 \| 0.058 \| \| 6 \| Assembly + Parasite taxon \| 2 \| 378.3 \| 1.54 \| 0.055 \| \| 7 \| Assembly + Functional group \| 2 \| 378.3 \| 1.63 \| 0.053 \| \| 8 \| Host taxon \| 1 \| 378.4 \| 1.7 \| 0.051 \| \| 9 \| Virulence \| 1 \| 378.5 \| 1.77 \| 0.049 \| \| 10 \| Host range \| 1 \| 378.7 \| 2.03 \| 0.043 \| \| 11 \| Reproduction \| 1 \| 378.8 \| 2.12 \| 0.042 \| \| 12 \| Duration \| 1 \| 378.8 \| 2.12 \| 0.041 \| \| 13 \| Setting \| 1 \| 378.8 \| 2.13 \| 0.041 \| \| 14 \| Functional group + Virulence \| 2 \| 378.9 \| 2.22 \| 0.039 \| \| 15 \| Assembly + Host taxon + Parasite taxon \| 3 \| 379.1 \| 2.39 \| 0.036 \| \| 16 \| Assembly + Functional group + Setting \| 3 \| 379.2 \| 2.46 \| 0.035 \| \| 17 \| Assembly + Host taxon \| 2 \| 379.2 \| 2.49 \| 0.035 \| \| 18 \| Host diversity + Functional group \| 2 \| 379.2 \| 2.52 \| 0.034 \| \| 19 \| Assembly + Functional group + Virulence \| 3 \| 379.3 \| 2.64 \| 0.032 \| \| 20 \| Assembly + Host diversity \| 2 \| 379.4 \| 2.66 \| 0.032 \| |
| **B. Conditional^*^ model-averaged coefficients.** The effect size statistic is Hedges’ *g.*   \| **Factor** \| **Levels**^†^ \| **Coefficient** \| **95% CI** \| **z** \| **p** \| **Importance** \| \| --- \| --- \| --- \| --- \| --- \| --- \| --- \| \| Intercept \|  \| -0.868 \| [-1.909,0.173] \| 1.635 \| 0.102 \|  \| \| Assembly \| Mating v. Combining \| 0.602 \| [-0.225,1.430] \| 1.427 \| 0.153 \| 0.49 \| \| Functional group \| Micro v. Macroparasite \| 0.261 \| [-0.136,0.658] \| 1.287 \| 0.198 \| 0.43 \| \| Parasite taxon \| Fungal v. Other \| 0.278 \| [-0.195,0.750] \| 1.152 \| 0.250 \| 0.37 \| \| Setting \| Lab v. Field \| 0.285 \| [-0.375,0.944] \| 0.846 \| 0.397 \| 0.33 \| \| Virulence \| Low v. High \| 0.298 \| [-0.355,0.952] \| 0.895 \| 0.371 \| 0.32 \| \| Host diversity \| High v. Low \| 0.144 \| [-0.189,0.476] \| 0.846 \| 0.398 \| 0.29 \| \| Host taxon \| Other v. Invertebrate \| 0.303 \| [-0.454,1.059] \| 0.784 \| 0.433 \| 0.29 \| \| Reproduction \| Other v. Outcrossing \| 0.015 \| [-0.859,0.888] \| 0.033 \| 0.974 \| 0.25 \| \| Duration \| Long v. Short \| 0.057 \| [-0.446,0.560] \| 0.223 \| 0.824 \| 0.24 \| \| Host range \| General v. Specific \| 0.032 \| [-0.305,0.369] \| 0.184 \| 0.854 \| 0.23 \| \| Parasitism metric \| Load v. Prevalence \| 0.090 \| [-0.217,0.398] \| 0.575 \| 0.565 \| 0.15 \| \|  \| Load v. Virulence \| 0.380 \| [-0.306,1.066] \| 1.087 \| 0.277 \|  \| |
|  |
| ^*^Conditional indicates that model averaging included only those models in which the factor of interest appeared.  ^†^The left-hand level of each factor indicates the reference level. |

| **Table S4: Analysis of context dependence in the subset of crop studies reporting standard deviations.** |
| --- |
| **A. Results of model selection analysis: all models**   \| **Rank** \| **Terms** \| **N Factors** \| **AICc** \| **ΔAIC** \| **Weight *w*** \| \| --- \| --- \| --- \| --- \| --- \| --- \| \| 1 \| Evenness + Inoculation mode \| 2 \| 974.0 \| 0.00 \| 0.233 \| \| 2 \| Evenness \| 1 \| 974.5 \| 0.54 \| 0.178 \| \| 3 \| Evenness + Inoculation mode + Parasitism metric \| 3 \| 974.6 \| 0.67 \| 0.167 \| \| 4 \| Evenness + Parasitism metric \| 2 \| 975.0 \| 1.04 \| 0.138 \| \| 5 \| Evenness + Inoculation mode + Parasite life cycle \| 3 \| 975.6 \| 1.64 \| 0.103 \| \| 6 \| Evenness + Parasite life cycle \| 2 \| 976.3 \| 2.36 \| 0.072 \| \| 7 \| Evenness + Inoculation mode + Parasite life cycle + Parasitism metric \| 4 \| 976.6 \| 2.66 \| 0.061 \| \| 8 \| Evenness + Parasite life cycle + Parasitism metric \| 3 \| 977.1 \| 3.17 \| 0.048 \| \| 9 \| Inoculation mode \| 1 \| 987.8 \| 13.85 \| 0.000 \| \| 10 \| Inoculation mode + Parasitism metric \| 2 \| 988.7 \| 14.73 \| 0.000 \| \| 11 \| Inoculation mode + Parasitism life cycle \| 2 \| 989.6 \| 15.57 \| 0.000 \| \| 12 \| Inoculation mode + Parasite life cycle + Parasitism metric \| 3 \| 990.7 \| 16.74 \| 0.000 \| \| 13 \| (intercept) \| 0 \| 992.4 \| 18.46 \| 0.000 \| \| 14 \| Parasitism metric \| 1 \| 993.3 \| 19.27 \| 0.000 \| \| 15 \| Parasite life cycle \| 1 \| 994.4 \| 20.40 \| 0.000 \| \| 16 \| Parasite life cycle + Parasitism metric \| 2 \| 995.4 \| 21.40 \| 0.000 \| |
| **B. Conditional^*^ model-averaged coefficients.** The effect size statistic is Hedges’ *g.*   \| **Factor** \| **Levels**^†^ \| **Coefficient** \| **95% CI** \| **z** \| **p** \| **Importance** \| \| --- \| --- \| --- \| --- \| --- \| --- \| --- \| \| Intercept \|  \| -2.995 \| [-4.586,-1.405] \| 3.691 \| <0.001 \|  \| \| Evenness \| Uneven v. Even \| 1.150 \| [0.638,1.661] \| 4.403 \| <0.001 \| 1.00 \| \| Inoculation mode \| Direct v. Passive \| 1.080 \| [0.208,1.952] \| 2.428 \| 0.015 \| 0.56 \| \| Parasitism metric \| Load v. Prevalence \| 0.668 \| [-0.369,1.706] \| 1.263 \| 0.207 \| 0.34 \| \| Parasite life cycle \| Simple v. Complex \| 0.844 \| [-1.678,3.365] \| 0.656 \| 0.512 \| 0.20 \| |
| ^*^Conditional indicates that model averaging included only those models in which the factor of interest appeared.  ^†^The left-hand level of each factor indicates the reference level. |

| **Table S5: Analysis of context dependence in the full collection of crop studies.** This analysis included all 55 crop studies. We calculated the weighted average effect size for levels of each factor across studies and across experiments, with confidence intervals obtained by bootstrapping. The effect size statistic used in analyses was *lnRR*, which we then converted to the response ratio (ratio of mean parasitism in high vs. low diversity treatments), shown here for ease of interpretation. |
| --- |
| \|  \|  \| *Across studies* \| \| *Across experiments* \| \| \| --- \| --- \| --- \| --- \| --- \| --- \| \| **Factor** \| **Level** \| **Response ratio** \| **95% CI** \| **Response ratio** \| **95% CI** \| \| Evenness^*^ \| Even \| 0.570 \| [0.416,0.724] \| 0.580 \| [0.489,0.688] \| \|  \| Uneven \| 0.585 \| [0.412,0.755] \| 0.261 \| [0.161,0.393] \| \| Inoculation mode^†^ \| Passive \| 0.549 \| [0.405,0.690] \| 0.483 \| [0.402,0.583] \| \|  \| Direct \| 0.602 \| [0.416,0.784] \| 0.532 \| [0.358,0.704] \| \| Poaceae \| Y \| 0.550 \| [0.415,0.693] \| 0.470 \| [0.388,0.563] \| \|  \| N \| 0.688 \| [0.668,0.793] \| 0.734 \| [0.667,0.793] \| \| Wheat \| Y \| 0.567 \| [0.403,0.734] \| 0.592 \| [0.390,0.819] \| \|  \| N \| 0.586 \| [0.425,0.739] \| 0.471 \| [0.391,0.565] \| |
|  |

^*^ We distinguished between experiments and studies that included only even mixtures vs. a combination of even and uneven mixtures. Hence, estimates from "uneven" studies may include a large number of effect sizes from even mixtures. In contrast, individual experiments were more likely to include only even or only uneven mixtures.

^†^ For inoculation mode, we distinguished between experiments and studies with passive exposure (natural) vs. direct, artificial inoculation. For the subset of eight studies reporting standard deviations, direct inoculation referred to artificial inoculation of all plants (i.e. spraying, studies=2) or of a subset of plants in the plot (studies=2). In the larger set of studies, direct inoculation more commonly entailed artificial inoculation of a subset of plants (studies = 8) or transplanting of infected plants into plots to serve as inocula (n=11); inoculation of all plants via spraying was less common (n=9).

| **Table S6: Analysis of context dependence in observational studies of non-crop systems.** |
| --- |
| **A. Results of model selection analysis: top 20 models**   \| **Rank** \| **Terms** \| **N Factors** \| **AICc** \| **ΔAIC** \| **Weight *w*** \| \| --- \| --- \| --- \| --- \| --- \| --- \| \| 1 \| IUCN status + Parasite life cycle \| 2 \| 113.1 \| 0.00 \| 0.065 \| \| 2 \| IUCN status + Parasite life cycle + Host taxon \| 3 \| 114.6 \| 1.52 \| 0.031 \| \| 3 \| IUCN status + Parasite life cycle + Island status \| 3 \| 114.7 \| 1.59 \| 0.029 \| \| 4 \| IUCN status + Parasite life cycle + Virulence \| 3 \| 114.9 \| 1.84 \| 0.026 \| \| 5 \| IUCN status + Parasite life cycle + Parasitism metric \| 3 \| 115.0 \| 1.93 \| 0.025 \| \| 6 \| IUCN status + Parasite life cycle + Host range \| 3 \| 115.1 \| 2.09 \| 0.023 \| \| 7 \| IUCN status + Parasite life cycle + Parasite taxon \| 3 \| 115.2 \| 2.14 \| 0.022 \| \| 8 \| IUCN status + Island status + Parasitism metric \| 3 \| 115.3 \| 2.21 \| 0.022 \| \| 9 \| IUCN status + Parasite life cycle + Reproduction \| 3 \| 115.3 \| 2.28 \| 0.021 \| \| 10 \| IUCN status + Parasite life cycle + Functional group \| 3 \| 115.5 \| 2.48 \| 0.019 \| \| 11 \| IUCN status + Parasite life cycle + Island status + Host taxon \| 4 \| 115.8 \| 2.75 \| 0.016 \| \| 12 \| Island status + Parasitism metric \| 2 \| 115.8 \| 2.77 \| 0.016 \| \| 13 \| IUCN status + Island status \| 2 \| 115.9 \| 2.86 \| 0.016 \| \| 14 \| IUCN status + Parasite life cycle + Island status + Parasitism metric \| 4 \| 116.1 \| 3.05 \| 0.014 \| \| 15 \| IUCN status + Parasite life cycle + Host taxon + Parasite taxon \| 4 \| 116.1 \| 3.08 \| 0.014 \| \| 16 \| Island status + Parasitism metric + Functional group \| 3 \| 116.2 \| 3.16 \| 0.013 \| \| 17 \| IUCN status \| 1 \| 116.3 \| 3.24 \| 0.013 \| \| 18 \| IUCN status + Island status + Parasitism metric + Functional group \| 4 \| 116.3 \| 3.25 \| 0.013 \| \| 19 \| IUCN status + Parasitism metric \| 2 \| 116.4 \| 3.32 \| 0.012 \| \| 20 \| Parasite life cycle + Island status \| 2 \| 116.4 \| 3.34 \| 0.012 \| |
| **B. Conditional^*^ model-averaged coefficients.** The effect size statistic used in analyses was Fisher’s *z*, which we then converted to the correlation coefficient *r*, shown here for ease of interpretation.   \| **Factor** \| **Levels**^†^ \| **Coefficient** \| **95% CI** \| **z** \| **p** \| **Importance** \| \| --- \| --- \| --- \| --- \| --- \| --- \| --- \| \| Intercept \|  \| -0.303 \| [-0.760,0.355] \| 0.895 \| 0.371 \|  \| \| IUCN status \| Not vs. Threatened \| -0.505 \| [-0.759,-0.118] \| 2.491 \| 0.013 \| 0.72 \| \| Parasite life cycle \| Simple vs. Complex \| 0.267 \| [0.002,0.498] \| 1.971 \| 0.049 \| 0.53 \| \| Island status \| Island vs. mainland \| 0.442 \| [-0.014,0.746] \| 1.904 \| 0.057 \| 0.52 \| \| Parasitism metric \| Load v. Prevalence \| 0.203 \| [-0.086,0.460] \| 1.382 \| 0.167 \| 0.33 \| \| Host range \| General v. Specific \| 0.147 \| [-0.185,0.448] \| 0.864 \| 0.388 \| 0.22 \| \| Virulence \| Low v. High \| 0.131 \| [-0.130,0.374] \| 0.984 \| 0.325 \| 0.21 \| \| Host taxon \| Other v. Invertebrate \| 0.124 \| [-0.177,0.404] \| 0.805 \| 0.421 \| 0.2 \| \| Functional group \| Micro v. Macroparasite \| 0.109 \| [-0.272,0.461] \| 0.554 \| 0.580 \| 0.18 \| \| Reproduction \| Other v. Outcrossing \| -0.111 \| [-0.410,0.209] \| 0.676 \| 0.499 \| 0.17 \| \| Parasite taxon \| Animal v. Other \| 0.020 \| [-0.285,0.321] \| 0.127 \| 0.899 \| 0.15 \| |
| ^*^Conditional indicates that model averaging included only those models in which the factor of interest appeared.  ^†^The left-hand level of each factor indicates the reference level. |

| **Table S7: Combined analysis of experimental and observational data.** This analysis included 460 effect sizes from 55 studies. Average Hedges' *g* for each study type are reported. |
| --- |
| \| **Factor** \| **Estimate** \| **95% CI** \| **z** \| **p** \| \| --- \| --- \| --- \| --- \| --- \| \| Experimental crop \| -1.662 \| [-2.295,-1.029] \| -5.147 \| <0.001 \| \| Experimental non-crop \| -0.472 \| [-0.878,-0.067] \| -2.282 \| 0.023 \| \| Observational non-crop \| -0.169 \| [-0.617,0.278] \| -0.742 \| 0.458 \| |

Supplemental Figures

| 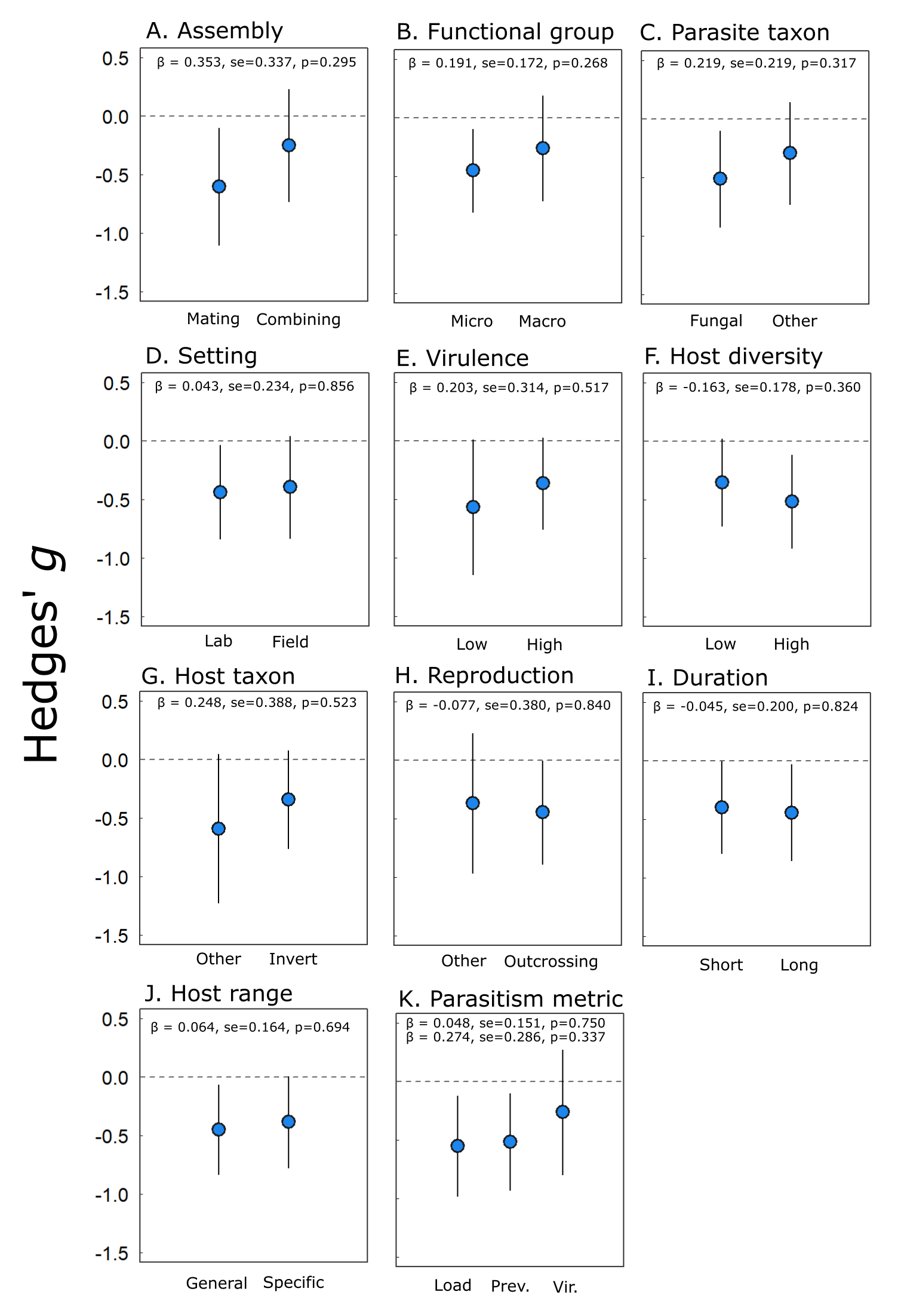 |
| --- |
| **Figure S1: Testing for context-dependence in experimental studies of non-crop systems.** Each panel shows the results of a model fit with a single contextual factor. For no factor did  factor levels differ significantly from one another. The results presented here differ from those in Table S3, which presents model-averaged coefficients over a 95% confidence set of candidate models accounting for many factors at once. |

| 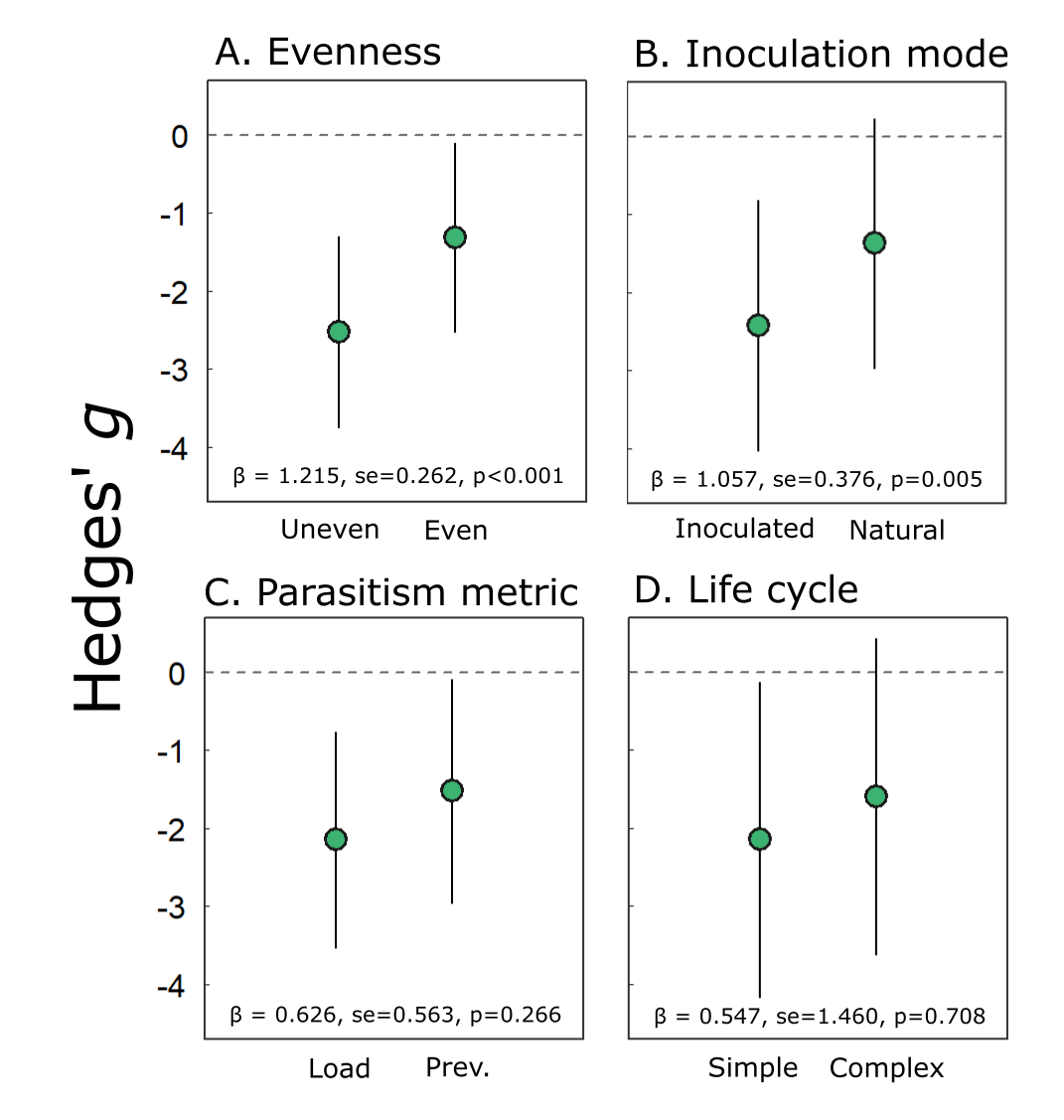 |
| --- |
| **Figure S2: Testing for context-dependence in experimental studies of crop systems.** This analysis included only the eight studies with standard deviations reported. Each panel shows the results of a model fit with a single contextual factor. The results presented here differ from those in Table S4, which presents model-averaged coefficients over a 95% confidence set of candidate models accounting for many factors at once. |

| 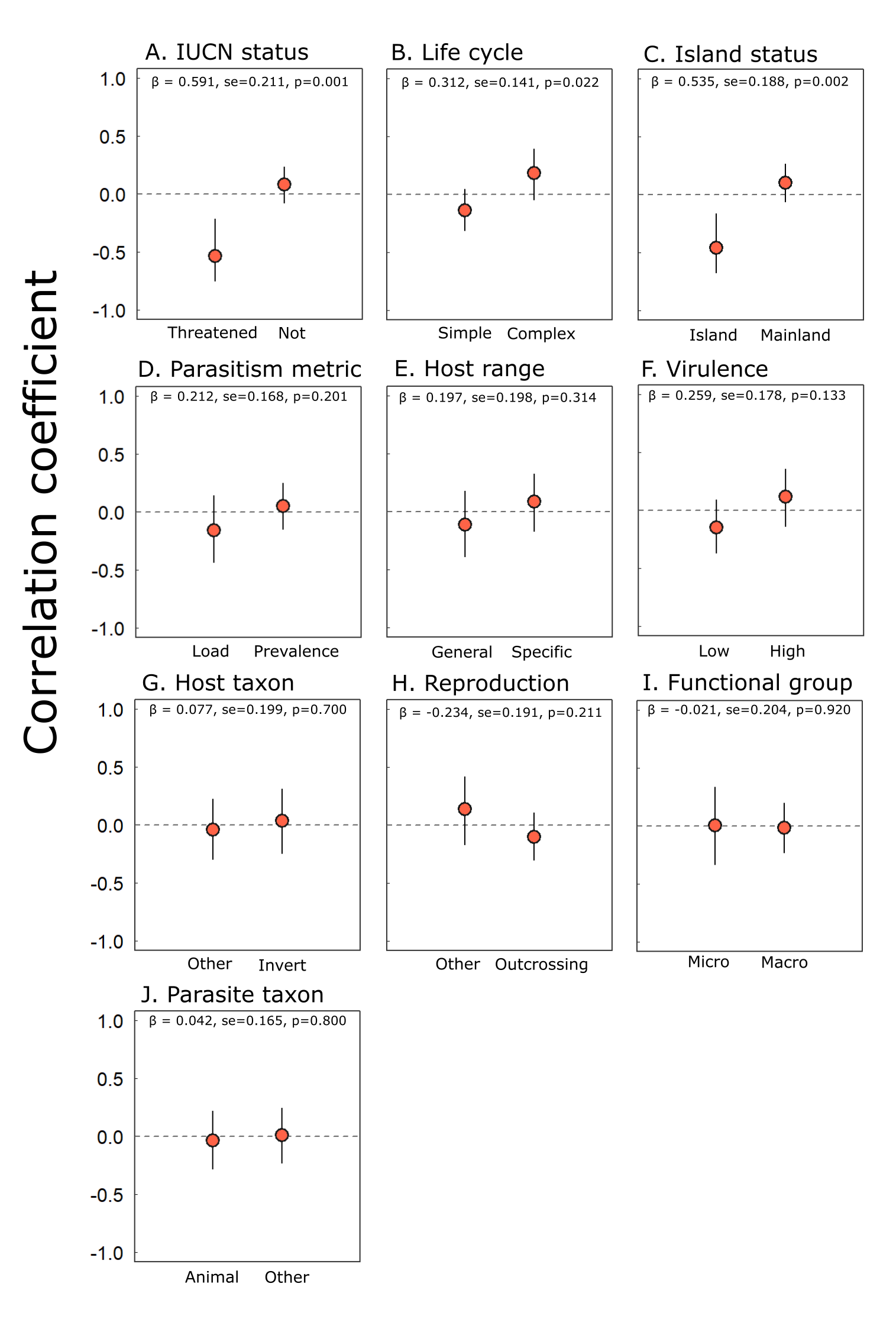 |
| --- |
| **Figure S3: Testing for context-dependence in observational studies of non-crop systems.** Each panel shows the results of a model fit with a single contextual factor. Plots and coefficients are presented in terms of the correlation coefficient *r* after conversion from Fisher’s *z*. The results presented here differ from those in Table S6, which presents model-averaged coefficients over a 95% confidence set of candidate models accounting for many factors at once. |

References in Supplement

Brütsch, T., A. Avril, and M. Chapuisat. 2017. No evidence for social immunity in co-founding queen associations. Scientific Reports 7:1-8.

Civitello, D. J., J. Cohen, H. Fatima, N. T. Halstead, J. Liriano, T. A. McMahon, C. N. Ortega et al. 2015. Biodiversity inhibits parasites: broad evidence for the dilution effect. Proceedings of the National Academy of Sciences 112:8667-8671.

COSEWIC. 2010. COSEWIC assessment and status report on the Atlantic Salmon Salmo salar (Nunavik population, Labrador population, Northeast Newfoundland population, South Newfoundland population, Southwest Newfoundland population, Northwest Newfoundland population, Quebec Eastern North Shore population, Quebec Western North Shore population, Anticosti Island population, Inner St. Lawrence population, Lake Ontario population, Gaspé-Southern Gulf of St. Lawrence population, Eastern Cape Breton population, Nova Scotia Southern Upland population, Inner Bay of Fundy population, Outer Bay of Fundy population) in Canada. Committee on the Status of Endangered Wildlife in Canada Ottawa:xlvii + 136 pp. ([www.sararegistry.gc.ca/status/status_e.cfm](http://www.sararegistry.gc.ca/status/status_e.cfm)).

Desai, S. D., and R. W. Currie. 2015. Genetic diversity within honey bee colonies affects pathogen load and relative virus levels in honey bees, *Apis mellifera* L. Behavioral Ecology and Sociobiology 69:1527-1541.

Dionne, M., K. M. Miller, J. J. Dodson, and L. Bernatchez. 2009. MHC standing genetic variation and pathogen resistance in wild Atlantic salmon. Philosophical Transactions of the Royal Society B: Biological Sciences 364:1555-1565.

Ekroth, A. K., C. Rafaluk-Mohr, and K. C. King. 2019. Host genetic diversity limits parasite success beyond agricultural systems: a meta-analysis. Proceedings of the Royal Society B 286:20191811.

Hargreaves, A. L., R. M. Germain, M. Bontrager, J. Persi, and A. L. Angert. 2020. Local adaptation to biotic interactions: A meta-analysis across latitudes. The American Naturalist 195:395-411.

Hughes, W., F. Ratnieks, and B. Oldroyd. 2008. Multiple paternity or multiple queens: two routes to greater intracolonial genetic diversity in the eusocial Hymenoptera. Journal of Evolutionary Biology 21:1090-1095.

Hughes, W. O., and J. J. Boomsma. 2004. Genetic diversity and disease resistance in leaf-cutting ant societies. Evolution 58:1251-1260.

IUCN. 2020. The IUCN Red List of Threatened Species, Version 2020-2. <https://www.iucnredlist.org>, Downloaded on 027 July 2020.

Lau, J., J. P. Ioannidis, N. Terrin, C. H. Schmid, and I. Olkin. 2006. The case of the misleading funnel plot. BMJ 333:597-600.

Nakagawa, S., and E. S. Santos. 2012. Methodological issues and advances in biological meta-analysis. Evolutionary Ecology 26:1253-1274.

Neumann, P., and R. Moritz. 2000. Testing genetic variance hypotheses for the evolution of polyandry in the honeybee (*Apis mellifera* L.). Insectes Sociaux 47:271-279.

Oxley, P. R., and B. P. Oldroyd. 2010. The genetic architecture of honeybee breeding, Pages 83-118 Advances in Insect Physiology, Elsevier.

Pagán, I., P. González-Jara, A. Moreno-Letelier, M. Rodelo-Urrego, A. Fraile, D. Pinero, and F. García-Arenal. 2012. Effect of biodiversity changes in disease risk: exploring disease emergence in a plant-virus system. PLoS Pathogens 8.

Queirós, J., J. Vicente, P. C. Alves, J. de la Fuente, and C. Gortazar. 2016. Tuberculosis, genetic diversity and fitness in the red deer, *Cervus elaphus*. Infection, Genetics and Evolution 43:203-212.

Reber, A., G. Castella, P. Christe, and M. Chapuisat. 2008. Experimentally increased group diversity improves disease resistance in an ant species. Ecology Letters 11:682-689.

Savage, A. E., C. G. Becker, and K. R. Zamudio. 2015. Linking genetic and environmental factors in amphibian disease risk. Evolutionary Applications 8:560-572.

Sherman, P. W., T. D. Seeley, and H. K. Reeve. 1988. Parasites, pathogens, and polyandry in social Hymenoptera. American Naturalist 131:602-610.

Sterne, J. A., A. J. Sutton, J. P. Ioannidis, N. Terrin, D. R. Jones, J. Lau, J. Carpenter et al. 2011. Recommendations for examining and interpreting funnel plot asymmetry in meta-analyses of randomised controlled trials. BMJ 343:d4002.

Uyeda, J. C., R. Zenil-Ferguson, and M. W. Pennell. 2018. Rethinking phylogenetic comparative methods. Systematic Biology 67:1091-1109.
